# Outcomes of Patients with Newly Diagnosed Cardiac Myxoma: A Retrospective Multicentric Study

**DOI:** 10.1155/2018/8320793

**Published:** 2018-02-06

**Authors:** Frantisek Nehaj, Juraj Sokol, Michal Mokan, Veronika Jankovicova, Frantisek Kovar, Marianna Kubaskova, Stanislav Mizera, Marian Mokan

**Affiliations:** ^1^First Department of Internal Medicine, Jessenius Faculty of Medicine in Martin, Comenius University in Bratislava, Kollarova 2, 036 59 Martin, Slovakia; ^2^Department of Haematology and Transfusion Medicine, National Centre of Haemostasis and Thrombosis, Jessenius Faculty of Medicine in Martin, Comenius University in Bratislava, Kollarova 2, 036 59 Martin, Slovakia; ^3^Central Slovakian Institute for Cardiac and Vascular Diseases, Cesta K Nemocnici 622/1, 974 01 Banska Bystrica, Slovakia; ^4^Department of Radiology, Jessenius Faculty of Medicine in Martin, Comenius University in Bratislava, Kollarova 2, 036 59 Martin, Slovakia; ^5^The National Institute of Cardiovascular Diseases, Pod Krasnou Horkou 1, 833 48 Bratislava 37, Slovakia

## Abstract

The patient database at the First Department of Internal Medicine in Martin, the Central Slovak Institute for Cardiac and Vascular Diseases in Banska Bystrica, and the National Slovak Institute of Cardiovascular Diseases in Bratislava was searched to identify patients with benign tumors of the heart seen during the 5-year period between 2011 and 2016. Forty-one patients with primary cardiac myxomas were identified and their medical records were reviewed for details pertaining to presenting symptoms, staging modalities, treatment approaches, and outcomes. Most of the studied patients were diagnosed with echocardiography (*n* = 35, 85%). The occurrence of the tumor was higher in the female population (*n* = 25, 61%). The most common presenting symptoms were dyspnoea (*n* = 17, 42%), chest pain (*n* = 3, 7%), or pain and paraesthesia of the limbs (*n* = 2, 5%). Acute embolic event due to embolization of tumor fragments resulted in cerebral stroke (*n* = 5, 12%). All patients were treated by resection. Only one comorbid patient died due to multiple-organ dysfunction syndrome two weeks after the resection. The most common postoperative complication was bleeding (*n* = 2, 5%) and infection (*n* = 2, 5%). The early diagnosis and appropriate treatment are often curative, with very low risk of recurrence. Postoperative survival is high.

## 1. Introduction

First recognition of a heart tumor is attributed to Columbus in 1559 [1], followed by Malpighi, who in 1666 wrote a dissertation entitled “De polypo cordis” [2]. Bahnson and Newman (1953) reported the earliest surgical approaches to myxomas by removing a myxoma from the right atrium via right anterior thoracotomy using a short period of caval obstruction at normothermia. Crafoord (1955) successfully excised a myxoma from left atrium using cardiopulmonary bypass [3]. Coates and Drake (1958) reported a successful excision of a right atrial myxoma [4]. Patients with a heart tumor have a wide range of symptoms. General symptoms include exhaustion, dyspnoea, syncope, and chest pain. They could cause symptoms by three mechanisms: intracardiac obstruction, systematic embolization of tumor fragments, or constitutional symptoms by unclear mechanisms [5]. Obstruction may restrict the blood flow into the heart, mimicking stenosis of the mitral or tricuspid valve. Malignant tumors are often manifested with weight loss, fever, night sweating, or haemorrhagic pericardial effusion. Malignant tumors that metastasize to the heart are nearly 50 times more common than tumors originating from the heart [6]. Tumors that originate from the heart are benign in approximately 75% and nearly 50% of these are myxomas [7]. Other types of primary benign tumors of the heart include lipomas, fibromas, haemangiomas, teratomas, and rhabdomyomas. The frequency of primary cardiac tumors is approximately 0.02%, corresponding to 200 tumors in 1 million autopsies, based on the data of 22 large autopsy series [8–11]. Myxomas most commonly arise from left atrium, usually from a stalk attached to the atrial septum, 75% in the left atrium, up to 20% in the right atrium, and around 8% in the ventricles. Peripheral embolization occurs in up to 30% of cases and most of them embolize to the central nervous system [12]. Because of the very rare occurrence of primary cardiac tumors, there are only few case series in the literature describing the epidemiology, presentation, and outcome of cardiac myxomas. The purpose of this retrospective study is to define and describe the characteristic, behaviour, and prognostic indicators of cardiac myxomas on a large group of patients.

## 2. Methods

Three cardiovascular centres participated in this study: the First Department of Internal Medicine in Martin, the Central Slovak Institute for Cardiac and Vascular Diseases in Banska Bystrica, and the National Slovak Institute of Cardiovascular Diseases in Bratislava. The patient-related data were obtained from medical records. Between 2011 and 2016, 41 patients were diagnosed with primary cardiac tumor. All tumors were benign known as myxomas, 39 were in left atrium (95%), and 2 were in the right atrium (5%). These patients were treated with surgical resection, which is associated with excellent long-term survival. We did not observed recurrence in the presented group of patients. Statistical Package for the Social Sciences (SPSS, Inc., Chicago, Illinois, USA) was used for analysis. Statistical analysis consisted of basic descriptive statistics. Kaplan-Meier curves were constructed to estimate the cumulative survival.

## 3. Results

The most common presenting symptoms were dyspnoea (*n* = 17, 42%), chest pain (*n* = 3, 7%), or pain and paraesthesia of the limbs (*n* = 2, 5%). Nonspecific symptoms such as weight loss or fever were uncommon. Acute embolic event as a first sign of cardiac myxoma occurred in eight patients (20%). Cerebral stroke was the most frequent (*n* = 5, 12%). Some of the cases of cardiac myxoma were diagnosed accidentally (*n* = 4, 10%). The majority of the patients were diagnosed by echocardiography (*n* = 35, 85%). Other useful diagnostic methods were computed tomography (*n* = 5, 12.5%) or histology (*n* = 1, 2.5%).

More than half of the patients had no valvular heart disease (*n* = 21, 51%). The most common valvular heart disease not caused by heart tumor was aortic stenosis (*n* = 8, 20%). Mitral stenosis, secondary to tumor prolapse, was relatively common in patients with left atrial myxoma. There was a higher incidence of mitral regurgitation in patients with left atrial tumors. The patients with the right atrial myxoma had no valvular disease. Pulmonary hypertension was diagnosed in 51% of the patients (*n* = 21), mostly due to pulmonary diseases. Cardiac myxomas varied in size, the mean height was 3.27 ± 1.57 cm, and the mean length was 2.91 ± 0.91 cm. General characteristics and the diagnostic method of cardiac myxoma are shown in Table 1 and all echocardiographic and hemodynamic characteristics are shown in Table 3.

Despite tumor mass inside the left atrium of the heart, the systolic function remained good. The ejection fraction of the left ventricle, which was less than 50%, was present in 29% cases (*n* = 12). According to functional classification known as New York Heart association (NYHA), approximately 80% (*n* = 33) of patients presented in NYHA II/III. The presenting and other characteristics of cardiac myxoma are shown in Table 2.

Cardiovascular risk factors included diabetes mellitus in 44% (*n* = 18), cerebral stroke history in 20% (*n* = 8), hypertension in 85% (*n* = 35), and smoking in 22% (*n* = 9). The coronary artery disease (CAD) was present in 15 patients (37%). All the patients with triple-vessel disease (*n* = 7, 17%) were treated by coronary artery bypass grafting (CABG). Patients with single and double vessel disease underwent coronary angioplasty (20%).

The occurrence of the tumor was higher in a female population (*n* = 25, 61%), than in male population (*n* = 16, 39%). There was no significant difference in the occurrence of a heart tumor based on body weight. Approximately 7% of all cardiac myxomas are associated with Carney complex, which has an increased risk of recurrence and is a genetic disorder characterized by an increased risk of several types of tumors [13–16]. None of the patients from this study presented with Carney complex.

## 4. Surgical Statistics

None of the cardiac tumors were treated conservatively. Coronary artery bypass grafting and aortic valve repair or replacement were the most common concomitant surgical procedure. The concomitant operation was done in 27% patients (*n* = 11), from which only 7% (*n* = 3) underwent valve procedure. The incidence of postoperative complications was 17% (*n* = 7) and included sternal infection, pneumonia, bleeding requiring reoperation, and renal and respiratory insufficiency. The most common complication was infection (*n* = 2) and bleeding (*n* = 2). The infection occurred in both patients with diabetes. In general, the postoperative healing process is slower and the risk of infection is higher in diabetic patients. The bleeding complication with drop in haemoglobin levels (125 → 68 g/L and 130 → 65 g/L), verified as severe anaemia, was observed in 2 patients. The renal and respiratory insufficiency occurred in one comorbid patient, who died because of multiple-organ dysfunction syndrome two weeks after resection.

The surgical characteristics with extracorporeal circulation time (ECC), cross-clamp time, and temperature are shown in Table 4.

## 5. Survival Analysis

**Figure 1:**
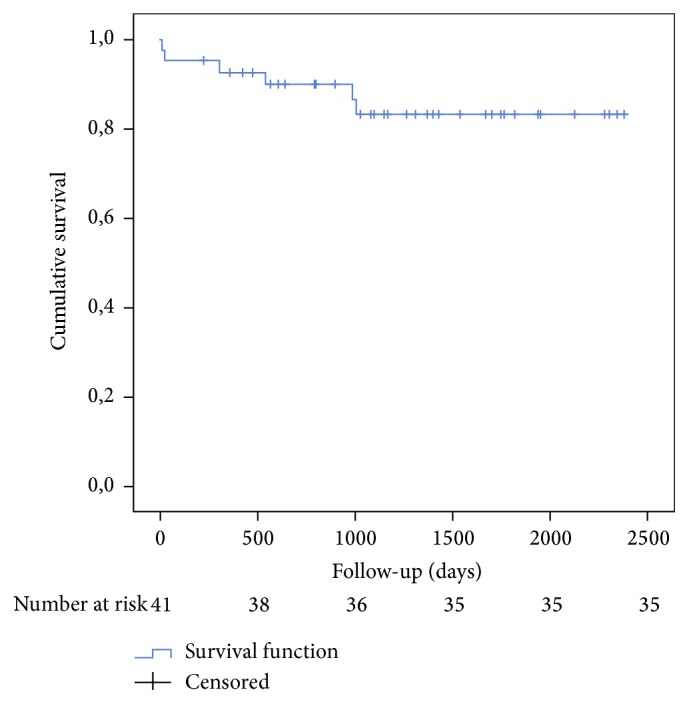
Kaplan-Maier curve of survival.

Duration of follow-up was counted from date of diagnosis to end of follow-up, defined as the date of the death or the date of last phone contact which was December 31, 2017. The median of follow-up was 221.53 ± 107.47 weeks (range 3–395 weeks). During follow-up, six patients died for an incidence rate 3.4 (95% CI 1.3–7.5) per 100 person-year. One patient died two weeks after surgery. The other five patients died for an average of 68 weeks after the resection; see Figure 1.

In this study, postoperative survival was not significantly different from age- and sex-matched individuals in the population. The mean age at tumor diagnosis was 61.78 ± 7.87 years. After discharge, all patients underwent medical examination with echocardiography. None of the patients from this study had recurrence of myxoma. Surgical en bloc resection with a margin of normal tissue, if anatomically feasible, is considered curative.

## 6. Discussion

Primary cardiac tumors are uncommon; cardiac myxoma has the highest incidence. The most significant predictor of cardiac tumors is histology. To illustrate how rare malignant primary cardiac tumors are, only 34 patients were identified at the Mayo Clinic between 1975 and 2007. All of them were sarcomas, and the most common histologic type was angiosarcoma (41%) [14]. Malignant tumors are more common in children. Cardiac myxomas occur in all age groups, most frequently in patients of ages between 40 and 60 [17, 18]. Women are more often diagnosed with cardiac myxoma also in this group of patients (*n* = 25, 61%).

In our retrospective study, all patients (*n* = 41) were treated with surgical resection. The postoperative survival was not different from age- and sex-matched individuals in the population with no recurrence of cardiac myxoma. The most common first symptom of the tumor was dyspnoea. Patients with presenting symptom such as peripheral embolism (*n* = 8, 19.5%) or patients who were asymptomatic (*n* = 4, 9.75%) were younger (53.87 ± 5.32) in comparison with the mean age of our study group. The majority of the patients were diagnosed by echocardiography. The ejection fraction of the left ventricle remains relatively good in most patients. Regarding cardiac mass inside the heart, mitral stenosis was common in patients with left atrial myxoma. Postoperative survival is high. Only one comorbid patient died due to multiple-organ dysfunction syndrome two weeks after the resection. The most common postoperative complication was bleeding and infection.

## 7. Conclusion

The most common first symptom of cardiac myxoma is dyspnoea. The majority of our patients were diagnosed by echocardiography. Prompt excision using cardiopulmonary bypass has been established as the only acceptable mode of treatment for these tumors. The surgeon must try to prevent fragmentation and intraoperative embolization. Nevertheless, the postoperative survival is high with a very low rate of complications.

## Figures and Tables

**Table 1:** General characteristics and diagnostic method of cardiac myxoma.

General characteristic	Number of patients or values
Mean age at the time of surgery, years ± SD (range)	61.78 ± 7.87 (48–81)
(i) >65 years	12
(ii) <65 years	29
Sex	
(i) Male	16
(ii) Female	25
BMI (kg/m2)	
(i) Normal weight (18.5–24.9)	13
(ii) Overweight (25.0–29.9)	16
(iii) Obese (30.0–39.9)	12
Location	
(i) Left atrium	39
(ii) Right atrium	2
Size of tumor	
(i) Tumor mean height in cm, ±SD (range)	3.27 ± 1.57 (0.90–7.50)
(ii) Tumor mean length in cm, ±SD (range)	2.91 ± 0.91 (1.20–5.00)
Carney complex	0
Diagnostic method	
(i) Echocardiography	35
(ii) Computed tomography	5
(iii) Histology	1

**Table 2:** Presenting and other characteristics of cardiac myxoma.

Presenting characteristics	Number of patients or value
Presenting sign or symptom	
Dyspnoea	17
Chest pain	3
Pain and paraesthesia of limbs	2
Palpitation	1
Peripheral embolism	
(a) Cerebral stroke	5
(b) Acute arterial occlusion	2
(c) Acute myocardial infarction	1
Atrial fibrillation	2
Syncope	1
Heart failure	1
Nonspecific symptom	
(a) Weight loss	1
(b) Fever	1
None	4
Rhythm	
(i) Sinus rhythm	33
(ii) Atrial fibrillation	8
Functional classification according to the New York Heart Association (NYHA)	
(i) I	6
(ii) II	25
(iii) III	8
(iv) IV	2
Risk factors	
(i) Diabetes mellitus	18
(ii) Arterial hypertension	35
(iii) Chronic obstructive disease of lungs	5
(iv) Smoking	9
Cerebral stroke history	8
Coronary artery disease	15
(i) Single-vessel disease	3
(ii) Double-vessel disease	5
(iii) Triple-vessel disease	7

**Table 3:** Echocardiographic and hemodynamic characteristics of cardiac myxoma.

Echocardiographic and hemodynamic characteristic	Number of patients or values
Valvular heart disease	
(i) Mitral regurgitation	4
(ii) Mitral stenosis	0
(iii) Aortic regurgitation	4
(iv) Aortic stenosis	8
(v) Tricuspid regurgitation	4
(vi) Pulmonary regurgitation	0
(vii) None	21
Ejection fraction	
(i) ≥50%	29
(ii) 30–49%	10
(iii) <30%	2
Pulmonary hypertension, mPAP, mmHg	
(i) Severe pulmonary hypertension >45 mmHg	4
(ii) Moderate pulmonary hypertension 36–45 mmHg	8
(iii) Mild pulmonary hypertension 25–35 mmHg	9
(iv) Normal	20
Carotid arteries	
(i) Hemodynamically significant stenosis ≥60%	0
(ii) Nonhemodynamically significant stenosis <60%	41

**Table 4:** Surgical characteristics and postoperative parameters of cardiac myxoma.

Operative parameters	Number of patients or values	
	Left atrium	Right atrium
Surgical procedure (resection)	39	2
Concomitant operations		
(i) Coronary artery bypass grafting	8	
(ii) Mitral valve procedure	1	
(iii) Aortic valve procedure	2	
(iv) Tricuspid valve procedure	0	
(v) Pulmonary valve procedure	0	
Extracorporeal time, mean time in minutes ± SD (range)	59.61 ± 30.917 (21–152)	
Cross-clamp time, mean time in minutes ± SD (range)	44.73 ± 17.574 (17–128)	
Cardiopulmonary bypass temperature, mean in degrees of Celsius ± SD (range)	34.15 ± 1.31 (29.7–36.0)	
Postoperative complications	7	
(i) Bleeding	2	
(ii) Renal insufficiency	1	
(iii) Pulmonary embolism	1	
(iv) Respiratory insufficiency	1	
(v) Infection	2	

## Abbreviations

CAD: Coronary artery disease
CABG: Coronary artery bypass grafting
ECC: Extracorporeal circulation time
NYHA: New York Heart Association
SPSS: Statistical Package for the Social Sciences.
